# Short-Term Modifications of Postural Balance Control in Young Healthy Subjects After Moderate Aquatic and Land Treadmill Running

**DOI:** 10.3389/fphys.2018.01681

**Published:** 2018-11-26

**Authors:** Alex Rizzato, Gerardo Bosco, Michael Benazzato, Antonio Paoli, Giulia Zorzetto, Attilio Carraro, Giuseppe Marcolin

**Affiliations:** ^1^Department of Biomedical Sciences, University of Padova, Padova, Italy; ^2^Department of Industrial Engineering, University of Padova, Padova, Italy; ^3^School of Human Movement Sciences, University of Padova, Padova, Italy

**Keywords:** stability, posture, bipedal stance, motor control, run

## Abstract

Postural balance control can be altered by land treadmill (LTM) running. This impairment seems to be related to a disturbance of vestibular and visual information. However, no studies are available on aquatic treadmill (ATM) running. The aim of the present study was to investigate the effect of running at moderate intensity over ATM and LTM on the postural balance control both with opened (OE) and closed (CE) eyes. Center of pressure (CoP) trajectory of 20 healthy subjects was collected on a dynamometric platform before and after a 20-min-long running on ATM and LTM at the same rate of perceived exertion (Borg's scale: 3/10). Heart rate (HR) was recorded every 30 s during running. Stabilogram diffusion analysis (SDA) and sample entropy (SampEn) were calculated to deepen motor control mechanisms. HR values were lower during ATM running with respect to LTM running (*p* < 0.01). A significant effect of the treadmill factor was detected in the OE condition for the sway path (*p* < 0.01; η_p_^2^ = 0.364; Power: 0.879), the sway area (*p* < 0.01; η_p_^2^ = 0.324; Power: 0.816), and the ML oscillations (*p* < 0.01; η_p_^2^ = 0.390; Power: 0.911) while an effect of the time factor was detected for the ellipse area (*p* < 0.05; η_p_^2^ = 0.213; Power: 0.576). However, the effect size for all the parameters ranged from 0.06 (trivial) to 0.48 (small). In the OE condition, the SDA highlighted a significant effect of the treadmill factor on all the short-term diffusion coefficients which negatively influenced the open loop motor control strategies. In the CE condition, SampEn analysis underlined a significant decrease of the CoP regularity after LTM running. Although slight modifications of the mechanisms involved in the postural balance control occurred, ATM and LTM moderate running did not seriously threaten postural balance performance. Therefore, the usage of ATM should be taken into account in all those situations where the well-known advantages of the aquatic environment are priorities.

## Introduction

The efficiency of the visual, vestibular, and proprioceptive system is fundamental in maintaining both static and dynamic postural stability. The center of pressure (CoP) displacement, derived from force platforms, is considered the most reliable output for postural balance control assessment. This approach provides good intra-session and inter-session reliability, especially designed to sway length, CoP X [medio-lateral (ML) sway], and CoP Y [anterior-posterior (AP) sway] parameters (Baldini et al., 2013). Moreover, CoP trajectory can be modeled as a fractional Brownian motion by means of the stabilogram diffusion analysis (SDA) revealing open loop and closed loop control mechanisms of posture (Collins and De Luca, 1993). Different studies considered SDA as a reliable method to rigorously explore postural balance control (Collins and De Luca, 1993; Doyle et al., 2008), thus it has extensively been employed over the last 25 years (Collins and De Luca, 1995; Derave et al., 2002; Laughton et al., 2003; Raymakers et al., 2005; Marcolin et al., 2016). Also, the time-development of the subsequent CoP trajectories is frequently considered as an indirect outcome of random and non-stationary fluctuations in the postural balance control throughout the employment of the sample entropy algorithm (SampEn) (Roerdink et al., 2006). This non-linear time series analysis quantifies the complexity of a time series in terms of regularity (Richman and Moorman, 2000). Besides postural balance control (Roerdink et al., 2006; Ramdani et al., 2009), the SampEn has been widely used in other fields as for heart rate variability (Al-Angari and Sahakian, 2007), neural respiratory signals (Chen et al., 2005), and electromyographical signals (Chen et al., 2009).

Postural balance performance can be altered by treadmill running. More in detail, Derave and colleagues demonstrated that running at moderate intensity impaired vestibular and visual information centers in young healthy subjects (Derave et al., 2002). Moreover, it has been proposed that exercise intensity affected postural balance control both when running is performed above (Nardone et al., 1997) and below (Derave et al., 2002) the anaerobic threshold. Also, prolonged running exercise influenced postural balance performance (Lepers et al., 1997; Degache et al., 2014; Marcolin et al., 2016). Recently, aquatic running has been considered as a surrogate to land running (Bressel et al., 2017). Running in an aquatic environment substantially reduced the impact on joints (Greene et al., 2009; Kanitz et al., 2015), elicited a greater oxygen uptake (Becker, 2009; Torres-Ronda and Del Alcázar, 2014), and reduced heart rate (HR) (Masumoto et al., 2005; Barbosa et al., 2007), due to blood-shift toward the intrathoracic vessels (Bosco et al., 2018). Studies comparing the effects on postural balance performance of the same physical exercises carried out in land and water condition are few and contradictory. Moreover, they investigated only the effect of walking training protocols in unhealthy subjects as stroke patients (Park et al., 2014; Lee et al., 2017). Only one study involved healthy subjects and demonstrated that a 30-min underwater walking, five times per week for 4 weeks, significantly improved the ML and the AP balance indexes (Lee and Kim, 2017).

To the best of our knowledge, no studies have evaluated the acute effect of aquatic running on postural balance control. Therefore, the main goal of our study was to investigate the acute effects of land and aquatic treadmill (ATM) running at a moderate intensity on the postural balance performance of young healthy subjects. Secondly, we adopted a stochastic modeling framework and a non-linear time series analysis to deepen the mechanisms involved in the postural balance control both with opened and closed eyes.

## Materials and methods

### Subjects

Twenty healthy subjects volunteered for the study (M = 6; F = 14; mean ± SD: 25 ± 5 years; 61 ± 11 kg; 1.67 ± 0.7 m). Subjects with no history of (i) orthopedic injuries in the last year, (ii) neurological pathologies, and (iii) sight, hearing or vestibular disorders were eligible for inclusion. All of the subjects gave their written informed consent and were free to renounce the study at any time.

### Experimental design

**Figure 1:**
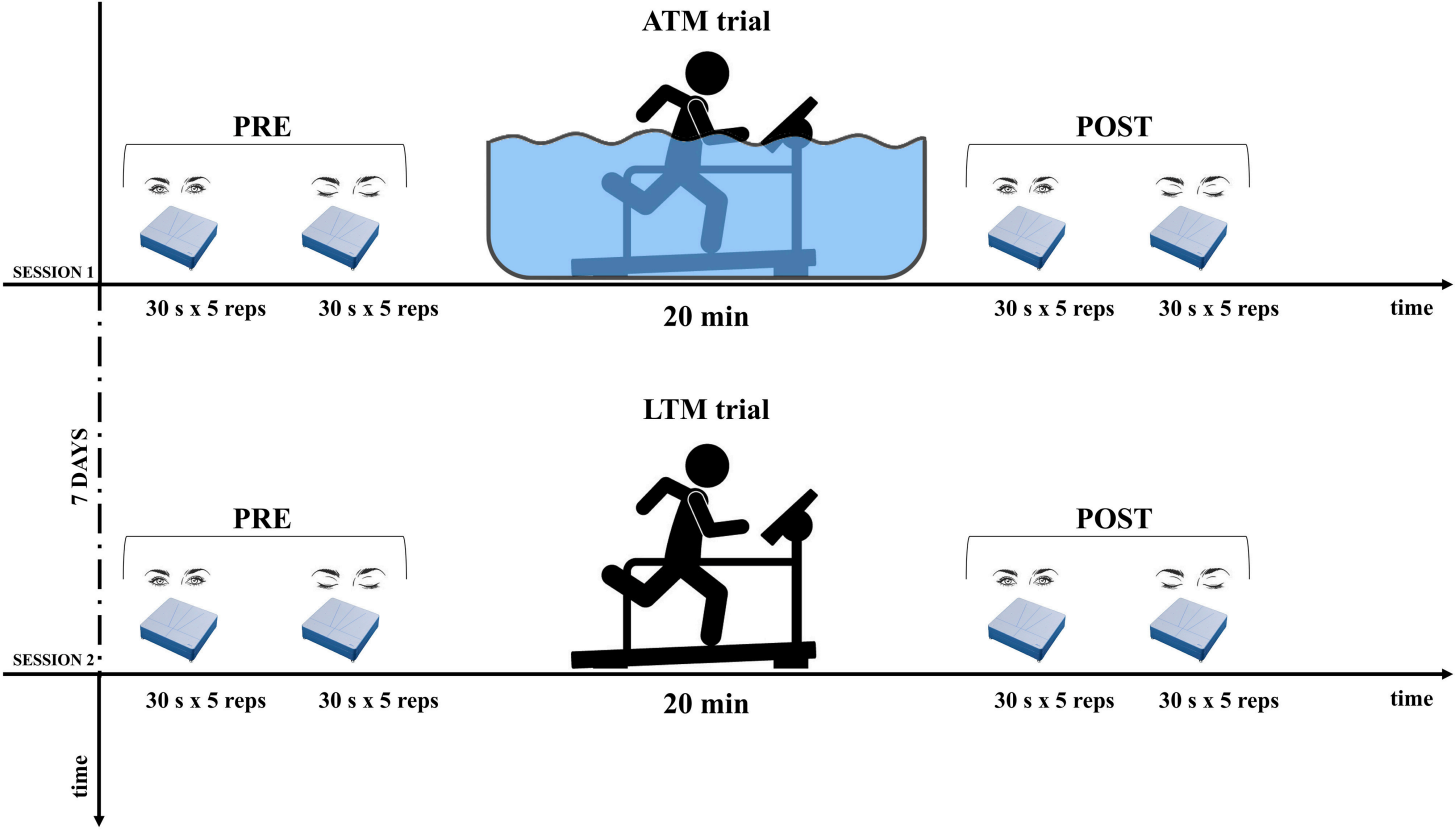
Experimental design. For both opened and closed eyes, postural balance assessment was performed before (PRE) and after (POST) aquatic-treadmill (ATM), and land-treadmill (LTM) trials. Sessions were administered following a cross-over design.

The experimental protocol adhered to the principles of the Declaration of Helsinki. Subjects involved in the study read and signed an informed consent and every precaution was taken to protect their privacy. All the subjects were informed about methods and aims of the study. The protocol was structured in 2 days as drafted in Figure 1.

We outlined a crossover design, in which subjects were randomly divided into two groups that received a sequence of two different exposures. The week before testing, researchers organized a familiarization session explaining in detail the scheduled program in order to guarantee its correct execution. Postural balance of each subject was tested on a dynamometric platform (RGMD S.p.a., Genova, Italy) before (PRE) and immediately after (POST) a 20-min-long moderate running on ATM (206060-Aqua Treadmill, Essenuoto, Italy) and LTM (Run-7410, Runner, Italy). Specifically, subjects had to perform the run guided by a Borg CR-10 scale (Borg, 1990) perceiving an intensity level corresponding to 3 (i.e., moderate), both in the ATM and LTM. The HR responses were monitored continuously by an HR chest belt (T31 coded™ transmitter, Polar Electro, Finland) and were recorded every 30 s. In the ATM running, subjects were partially-immersed up to the level of the xiphoid process. Water temperature was 27 ± 1°C.

The postural balance tests consisted on holding the same static upright position, both with opened eyes (OE) and closed eyes (CE). Subjects were instructed to stand with extended legs and arms naturally positioned along their sides. In the OE condition, they were asked to gaze a thin red line vertically placed on a white wall in front of them, at a distance of 80 cm. Test duration was 30 s according to Scoppa et al. (2013). Subjects were asked to perform five trials in the OE condition and five trials in the CE condition (Figure 1).

### Data analysis

CoP trajectory was collected at 100 Hz. The classical stabilometric parameters calculated from the CoP were: the area of the confidence ellipse [mm^2^], the sway path [mm/sec], the sway area [mm^2^/sec], the maximal oscillation in the ML and AP direction [mm], (Marcolin et al., 2016). The analysis was performed for each subject, extracting data from each trial concerning both OE and CE in the ATM and LTM conditions.

#### Stabilogram diffusion analysis

The SDA was performed in accord with Collins and colleagues (Collins and De Luca, 1993). The equation below calculates the mean square displacement <Δ*x*^2^> as a function of time interval Δ*t* (spanning *m* data intervals) for a CoP trajectory of *N* data point. The same equation was employed for < Δ*y*^2^> and < Δ*x*^2^>.

$$<\Delta x^{2}{>}_{\Delta t}=\frac{\sum_{i=l}^{N-m}{\left(\Delta x_{i}\right)}^{2}}{\left(N-m\right)}$$

The incremental values of Δ*t* ranged from 0.01 to 10 s with a step of 0.01 s. The plot of the mean square displacements vs. Δ*t* represented the stabilogram diffusion plots from whom we calculated the diffusion coefficients as presented elsewhere (Marcolin et al., 2016). Diffusion coefficients (*Df* ) were considered because they reflect the level of stochastic activity of the CoP, thus they represented an index of postural instability along the *x* axis (*Dfx*), the *y* axis (*Dfy*) or the combination of the two (*Dfr*^2^) (Collins and De Luca, 1993). The analysis tool was developed with MATLAB R2016b (The MathWorks, Inc., MA, USA).

#### Sample entropy

The SampEn gives a quantification of the entropy of time series, namely the regularity of the time series. The higher the SampEn value, the more irregular the time series (Richman and Moorman, 2000). Given an N-points time series [*x*_1_, *x*_2_, …, *x*_*N*_] with zero mean and unit variance, the first step of the SampEn algorithm consists on dividing the time series into *N* − *m* + 1 template vectors of length *m*:

$$\begin{array}{l} y_{i}\left(m\right)=\left[x_{i},x_{i+1},\ldots ,x_{i+m-1}\right]\text{ with}i=1,2,\ldots ,N-m+1 \end{array}$$

Then, the Chebyshev distance is computed between each template vector *y*_*i*_*(m)* and all the other *y*_*j*_*(m)* templates, *j* = 1, 2, …, *N-m*+1*, j* ! = *i*. Every time the distance between *y*_*i*_ and another template vector *y*_*j*_ is less than a radius *r*, this is called a match. Note that self-comparisons are not considered, to avoid self-matches. Let *B*_*i*_*(r)* be *(N-m*+1*)*^−1^ times the number of matches found for the generic template vector *y*_*i*_. Then, the following quantity is defined:

$$\begin{array}{l} B^{m}\left(r\right)=\frac{1}{N-m}\sum_{i=1}^{N-m}B_{i}\left(r\right). \end{array}$$

It represents the probability of finding a match between sequences of *m* consecutive points. The procedure is then repeated considering template vectors of length *m*+1 from the same time series, defining the quantity:

$$\begin{array}{l} A^{m}\left(r\right)=\frac{1}{N-m}\overset{N-m}{\underset{i=1}{\sum }}A_{i}\left(r\right) \end{array}$$

where *A*_*i*_*(r)* is *(N-m*+1*)*^−1^ times the number of matches found for the generic template vector *y*_*i*_ (of length *m*+1). *Am(r)* is then the probability of finding a match between sequences of *m*+1 consecutive points. The quantity

$$\begin{array}{l} CP\left(m,r\right)=\frac{A^{m}\left(r\right)}{B^{m}\left(r\right)} \end{array}$$

is the conditional probability that two sequences within a tolerance *r* for *m* points remain within *r* of each other at the next point (Richman and Moorman, 2000). The SampEn is defined as:

$$\begin{array}{l} \text{SampEn}\left(m,r\right)=\underset{N\to \infty }{\lim}\left[-\ln\;CP\left(m,r\right)\right] \end{array}$$

which is estimated as

$$\begin{array}{l} \text{SampEn}\left(m,r,N\right)=-\ln\;CP\left(m,r\right) \end{array}$$

The SampEn is the negative logarithm of the *CP(m,r)* probability; so, the more regular the signal, the higher the probability, the lower the SampEn, and vice versa.

Since the actual value of the SampEn requires a time series to have an infinite number of points, the value in the last equation is only an estimation of the SampEn value. An estimation of the variance of the *CP(m,r)* probability is (Lake et al., 2002):

$$\begin{array}{lll} \sigma_{CP}^{2}\left(m,r\right) & = & \frac{CP\left(m,r\right)\left[1-CP\left(m,r\right)\right]}{B\left(r\right)}\\ +\frac{1}{B{\left(r\right)}^{2}}\left[K_{A}-K_{B}\cdot CP{\left(m,r\right)}^{2}\right] \end{array}$$

where *B*(*r*) is defined as

$$\begin{array}{l} B\left(r\right)=\frac{\left(N-m+1\right)\left(N-m\right)}{2}B^{m}\left(r\right) \end{array}$$

*K*_*A*_ and *K*_*B*_ are the numbers of the overlapping matching template vectors of length *m* + 1 and *m*, respectively. Lake et al. (2002) suggested to fit the data with a standard *AR* model to choose the value of *m* and then select the value of *r* to minimize the quantity:

$$\begin{array}{l} Q\left(m,r\right)=\text{max}\left(\frac{\sigma_{CP}\left(m,r\right)}{CP\left(m,r\right)},\frac{\sigma_{CP}\left(m,r\right)}{-\log\left[CP\left(m,r\right)\right]CP\left(m,r\right)}\right) \end{array}$$

that is the maximum relative error of SampEn and of the *CP* estimate (Lake et al., 2002).

**Figure 2:**
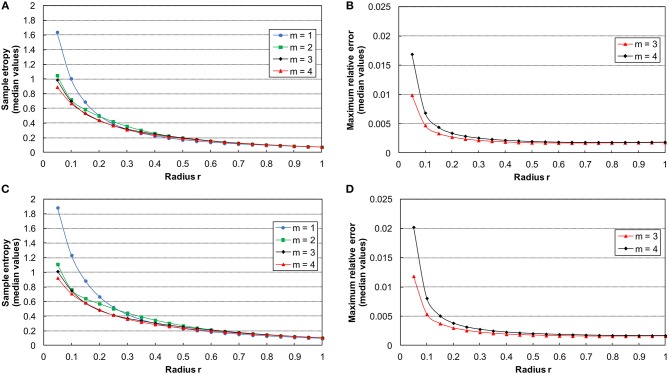
On the left side: curves of the median sample entropy estimation as function of *r* and *m* = 1–4 for ML **(A)** and AP **(C)** time series. On the right side: curves of the median of the maximum relative error for ML **(B)** and AP **(D)** time series with *m* = 3 and *m* = 4.

In the present study, the proper values of *m* and *r* for the SampEn computation followed the procedure presented elsewhere (Ramdani et al., 2009). Therefore, we randomly chose the 60% of both OE and CE increment time series. We calculated the median SampEn for *m* = 1, 2, 3, 4, and *r* from 0.05 to 1 with a step of 0.05 (Figures 2A,B) (Ramdani et al., 2009). Since the curves tend to converge for *m* ≥ 3, the maximum relative error *Q(m,r)* has been computed for *m* = 3, 4 (Figures 2C,D). Hence, we selected the *m* = 3 error curve, being the lowest one. Finally, we chose *r* = 0.35 as starting from this value the error curve reaches a steady state. The SampEn analysis tool was developed with MATLAB R2016b (The MathWorks, Inc., MA, USA).

### Statistical analysis

For each parameter, the mean value of the five trials performed by each subject was employed for statistical analysis. D'Agostino-Pearson test was employed to check data normality distribution. Paired *t*-test was employed to assess HR differences between ATM and LTM running. For both OE and CE condition a two-way ANOVA for repeated measures (time factor × treadmill factor) was performed to investigate dependent variables. Significant level for differences was set to *p* < 0.05. When *F*-value showed interactions between the factors, a *t*-test was used for pair-wise comparisons. Data analysis was performed using the software packages IBM SPSS Statistics for Windows (Version 24.0. Armonk, NY: IBM Corp). The effect size was calculated with G Power 3.1.5 (Faul et al., 2007). The magnitude of the effect size followed Cohen's guidelines (Cohen, 1988) and was interpreted as follow 0.00–0.19: trivial; 0.20–0.59: small; 0.60–1.19: moderate; 1.20–1.99: large and >2.00: very large (Hopkins et al., 2009).

## Results

All of the subjects successfully completed the study. The HR values recorded were significantly lower (*p* < 0.01; Effect Size: 0.75; Power: 0.89) in the ATM running (mean ± SD: 134.90 ± 13.30 b·min^−1^) with respect to the LTM running (mean ± SD: 145.80 ± 15.45 b·min^−1^). In detail, we observed a decrease in HR mean values from LTM to ATM running of 11 b·min^−1^ (−7.5%).

For the sake of clarity, postural balance results will be presented in separate subheadings as follows: classical postural parameters, SDA, and SampEn.

### Classical postural parameters

Tables 1, 2 report results collected before and after ATM and LTM running. In the OE condition a significant main effect of the treadmill factor was detected for the sway path (*p* < 0.01; η_p_^2^ = 0.364; Power: 0.879), the sway area (*p* < 0.01; η_p_^2^ = 0.324; Power: 0.816), and the ML oscillations (*p* < 0.01; η_p_^2^ = 0.390; Power: 0.911). Furthermore, a significant main effect of time factor was observed for the ellipse area (*p* < 0.05; η_p_^2^ = 0.213; Power: 0.576) and the ML oscillations (*p* < 0.01; η_p_^2^ = 0.299; Power: 0.760). In the CE condition, the analysis of variance did not show any statistically significant difference for all the classical parameters analyzed.

**Table 1:** Classical postural parameters: comparison between the PRE and the POST values in the OE condition.

	**ATM–OE**					**LTM–OE**				
	**PRE**	**POST**	**Δ**	**Δ95%CI**	**ES**	**PRE**	**POST**	**Δ**	**Δ95%CI**	**ES**
Ellipse area [mm^2^]	66.03 ± 29.07	71.61 ± 35.32	5.58 ± 6.24	[2.85, 8.31]	0.17	55.24 ± 22.69	70.02 ± 37.65	14.78 ± 14.96	[8.22, 21.30]	0.45
Sway path [mm/s]	7.80 ± 2.04	8.01 ± 2.75	0.20 ± 0.71	[−0.11, 0.51]	0.08	6.69 ± 1.37	7.64 ± 2.95	0.95 ± 1.58	[0.26, 1.64]	0.37
Sway area [mm^2^/s]	7.56 ± 3.32	8.34 ± 4.93	0.78 ± 1.61	[0.07, 1.49]	0.18	5.91 ± 1.90	7.83 ± 4.61	1.92 ± 2.71	[0.73, 3.11]	0.48
AP oscillations [mm]	13.65 ± 3.12	13.46 ± 3.08	−0.19 ± 0.04	[−0.21, −0.17]	0.06	13.26 ± 2.38	13.89 ± 3.27	0.63 ± 0.89	[0.24, 1.02]	0.21
ML oscillations [mm]	10.18 ± 3.05	11.23 ± 4.03	1.05 ± 0.98	[0.62, 1.48]	0.29	8.93 ± 2.18	10.11 ± 3.38	1.18 ± 1.20	[0.65, 1.71]	0.40

*Data are presented as mean ± standard deviation. Δ = difference between PRE and POST values; Δ95% CI = 95% Confidence interval of the differences between PRE and POST values; ES: effect size*.

**Table 2:** Classical postural parameters: comparison between the PRE and the POST values in the CE condition.

	**ATM–CE**					**LTM–CE**				
	**PRE**	**POST**	**Δ**	**Δ95%CI**	**ES**	**PRE**	**POST**	**Δ**	**Δ95%CI**	**ES**
Ellipse area [mm^2^]	99.93 ± 61.27	104.4 ± 64.96	4.47 ± 3.68	[2.86, 6.08]	0.07	92.19 ± 41.48	112.73 ± 80.15	20.55 ± 38.67	[3.65, 37.50]	0.29
Sway path [mm/s]	10.50 ± 3.34	9.83 ± 3.56	−0.67 ± 0.22	[−0.77, −0.57]	0.19	9.55 ± 2.91	9.81 ± 3.90	0.25 ± 0.99	[−0.18, 0.68]	0.07
Sway area [mm^2^/s]	12.38 ± 7.06	12.96 ± 8.27	0.57 ± 1.21	[0.04, 1.10]	0.07	10.85 ± 5.38	12.44 ± 7.93	1.59 ± 2.55	[0.47, 2.71]	0.23
AP Oscillations [mm]	17.31 ± 5.20	16.77 ± 5.46	−0.54 ± 0.23	[−0.64, −0.44]	0.10	16.89 ± 3.63	17.43 ± 4.21	0.54 ± 0.58	[0.29, 0.79]	0.14
ML oscillations [mm]	12.90 ± 4.60	13.38 ± 5.00	0.48 ± 0.40	[0.31, 0.66]	0.10	12.32 ± 3.70	13.59 ± 5.16	1.27 ± 1.45	[0.63, 1.91]	0.27

*Data are presented as mean ± standard deviation. Δ = difference between PRE and POST values; Δ95% CI = 95% Confidence interval of the differences between PRE and POST values; ES: effect size*.

### Stabilogram diffusion analysis

Tables 3, 4 report the SDA results before and after ATM and LTM running. In the OE condition, the two-way ANOVA for repeated measures showed a significant main effect of the treadmill factor for the *Dfxs* (*p* < 0.0001; η_p_^2^: 0.573; Power: 0.998), *Dfys* (*p* < 0.01; η_p_^2^: 0.326; Power: 0.819), and *Dfr*^2^*s* (*p* < 0.0001; η_p_^2^: 0.506; Power: 0.987). Moreover, a significant main effect of the time factor was observed in the *Dfxl* (*p* < 0.05; η_p_^2^: 0.279; Power: 0.730). In the CE condition a significant main effect of the treadmill factor was detected only for the *Dfxs* (*p* < 0.05; η_p_^2^: 0.258; Power: 0.685). Finally a main effect of time factor was registered in the *Dfr*^2^*l* (*p* < 0.05; η_p_^2^: 0.195; Power: 0.530).

**Table 3:** Stabilogram diffusion analysis results in the OE condition.

	**ATM–OE**					**LTM–OE**				
	**PRE**	**POST**	**Δ**	**Δ95%CI**	**ES**	**PRE**	**POST**	**Δ**	**Δ95%CI**	**ES**
*Dfsr^2^*	7.69 ± 3.50	8.04 ± 5.01	0.35 ± 3.20	[−1.05, 1.75]	0.08	5.64 ± 2.47	6.57 ± 3.73	0.93 ± 2.39	[−0.12, 1.98]	0.38
*Dflr^2^*	1.37 ± 0.70	1.46 ± 0.76	0.08 ± 0.58	[−0.17, 0.33]	0.12	1.38 ± 0.81	1.58 ± 0.76	0.19 ± 1.05	[−0.27, 0.65]	0.25
*Dfxs*	3.39 ± 2.12	3.31 ± 2.21	−0.07 ± 1.50	[−0.73, 0.59]	0.04	2.33 ± 1.32	2.41 ± 1.70	0.07 ± 1.11	[−0.42, 0.56]	0.05
*Dfxl*	0.40 ± 0.24	0.53 ± 0.47	0.13 ± 0.34	[−0.02, 0.28]	0.32	0.34 ± 0.18	0.54 ± 0.37	0.20 ± 0.37	[0.04, 0.36]	0.62
*Dfys*	4.30 ± 1.85	4.73 ± 2.99	0.43 ± 2.10	[−0.49, 1.35]	0.16	3.30 ± 1.33	4.15 ± 2.31	0.85 ± 1.64	[0.13, 1.57]	0.42
*Dfyl*	0.97 ± 0.59	0.92 ± 0.51	−0.04 ± 0.60	[−0.30, 0.22]	0.09	1.03 ± 0.68	1.03 ± 0.60	−0.002 ± 0.84	[−0.37, 0.37]	0.00

*Data are presented as mean ± standard deviation. Δ = difference between PRE and POST values; Δ95% CI = 95% Confidence interval of the differences between PRE and POST values; ES: Effect size*.

**Table 4:** Stabilogram diffusion analysis results in the CE condition.

	**ATM–CE**					**LTM–CE**				
	**PRE**	**POST**	**Δ**	**Δ95%CI**	**ES**	**PRE**	**POST**	**Δ**	**Δ95%CI**	**ES**
*Dfsr^2^*	14.82 ± 9.33	13.33 ± 8.86	−1.48 ± 5.79	[−1.06, 4.02]	0.16	12.66 ± 6.89	12.70 ± 8.36	0.04 ± 5.17	[−2.23, 2.31]	0.05
*Dflr^2^*	1.56 ± 1.18	1.91 ± 1.43	0.35 ± 0.95	[−0.07, 0.77]	0.26	1.42 ± 0.65	2.12 ± 1.72	0.70 ± 1.72	[−0.05, 1.45]	0.46
*Dfxs*	5.18 ± 3.54	4.45 ± 2.84	−0.72 ± 2.22	[−1.69, 0.25]	0.22	4.12 ± 2.28	4.07 ± 3.05	−0.05 ± 1.95	[−0.91, 0.81]	0.02
*Dfxl*	0.58 ± 0.59	0.72 ± 0.76	0.14 ± 0.73	[−0.18, 0.46]	0.20	0.43 ± 0.30	0.91 ± 1.14	0.48 ± 1.06	[0.02, 0.95]	0.47
*Dfys*	9.63 ± 6.39	8.87 ± 6.30	−0.75 ± 4.38	[−1.17, 2.67]	0.12	8.53 ± 4.93	8.63 ± 5.69	0.10 ± 3.55	[−1.46, 1.66]	0.02
*Dfyl*	0.98 ± 0.71	1.19 ± 0.88	0.20 ± 0.61	[−0.07, 0.47]	0.26	0.98 ± 0.56	1.20 ± 0.71	0.21 ± 0.79	[−0.14, 0.56]	0.34

*Data are presented as mean ± standard deviation. Δ = difference between PRE and POST values; Δ95% CI = 95% Confidence interval of the differences between PRE and POST values; ES: effect size*.

### Sample entropy

Tables 5, 6 show the AP and ML SampEn results. Specifically, in the OE condition there were no significant main effects or interactions both for the AP and ML SampEn. Conversely in the CE condition the two-way ANOVA for repeated measures showed a significant main effect of the time factor (*p* < 0.05; η_p_^2^: 0.292; Power: 0.757) considering the AP SampEn. Moreover, two significant interactions (time × treadmill) were detected in ML (*p* < 0.05; η_p_^2^: 0.283; Power: 0.738) and AP SampEn (*p* < 0.01; η_p_^2^: 0.315; Power: 0.801). The subsequent *t*-test comparing the PRE and POST condition revealed a significant decrease of the COP regularity considering the ML axis (*p* < 0.05; Effect Size: 0.33; Power: 0.16) and AP axis (*p* < 0.001; Effect Size: 0.46; Power: 0.06) only in the LTM running.

**Table 5:** Sample entropy results in the OE condition.

	**ATM–OE**					**LTM–OA**				
	**PRE**	**POST**	**Δ**	**Δ95%CI**	**ES**	**PRE**	**POST**	**Δ**	**Δ95%CI**	**ES**
ML	0.29 ± 0.06	0.30 ± 0.06	0.001 ± 0.03	[−0.012, 0.014]	0.17	0.30 ± 0.06	0.31 ± 0.06	0.01 ± 0.04	[−0.008, 0.028]	0.17
AP	0.35 ± 0.07	0.35 ± 0.07	0.007 ± 0.03	[−0.006, 0.020]	0.00	0.34 ± 0.06	0.35 ± 0.07	0.01 ± 0.02	[0.001, 0.019]	0.15

*Data are presented as mean ± standard deviation. Δ = difference between PRE and POST values; Δ95% CI = 95% Confidence interval of the differences between PRE and POST values; ES: effect size*.

**Table 6:** Sample entropy results in the CE condition.

	**ATM–CE**					**LTM–CE**				
	**PRE**	**POST**	**Δ**	**Δ95%CI**	**ES**	**PRE**	**POST**	**Δ**	**Δ95%CI**	**ES**
ML	0.28 ± 0.06	0.28 ± 0.07	−0.0002 ± 0.03	[−0.013, 0.013]	0.00	0.27 ± 0.04	0.29 ± 0.07	0.02 ± 0.04	[0.003, 0.038]	0.33
AP	0.32 ± 0.07	0.32 ± 0.06	0.003 ± 0.03	[−0.010, 0.016]	0.00	0.30 ± 0.06	0.33 ± 0.07	0.02 ± 0.03	[0.007, 0.033]	0.46

*Data are presented as mean ± standard deviation. Δ = difference between PRE and POST values; Δ95% CI = 95% Confidence interval of the differences between PRE and POST values; ES: effect size*.

## Discussion

A worsening of postural balance control seems to be related to treadmill running, due to a disturbance of vestibular and visual information (Lepers et al., 1997; Derave et al., 2002). Moreover, postural balance performance has been demonstrated to be influenced by prolonged running (Marcolin et al., 2016). Nonetheless, treadmill is a widely employed apparatus that is used in training as well as in rehabilitation programs. Similarly, the ATM became recognized for its numerous advantages, notably for reducing the force of impact on the joints (Greene et al., 2009; Kanitz et al., 2015). Moreover, water exercise has beneficial effects for the cardiovascular response (Barbosa et al., 2009). Specifically, it has been shown that HR was significantly lower when exercising with immersion to the breast, as compared to on land and with immersion to the hip (Barbosa et al., 2007). Additionally, Masumoto and colleagues reported a significant decrease of HR mean values from land-based to water exercises of 7.3 b·min^−1^ (Masumoto et al., 2005). Our HR results were in agreement with the above studies highlighting lower HR values in the ATM running. Surprisingly, although there is a high rate of employment of both ATM and LTM for recreational and rehabilitation purposes, there are no studies in the literature that compare their acute effects on postural balance.

### Postural balance performance

In the OE condition, classical postural parameters showed an effect of the time factor on the ellipse area. Since the smaller the surface of the ellipse the better the postural performance (Paillard and Noé, 2015), results of our study showed that this performance is more influenced by the running time than by the type of treadmill. Conversely, the COP sway path and sway area are representative of the number of continuous corrections adopted by the subject within the ellipse giving information on the efficiency of the postural balance control. Our results showed that in the OE condition only the type of treadmill influenced these two parameters. In the CE condition, no main effects or interactions were reached for the classical postural parameters analyzed. These results are not unexpected since it has already been demonstrated that treadmill exercise negatively affects visual contribution to static postural stability (Derave et al., 2002). Moreover, a prolonged running worsened classical stabilometric parameters when the postural test was performed with OE respect to CE (Marcolin et al., 2016). The interpretation of the statistical significances has to take into account the size of the differences in the PRE-POST comparison. Indeed, ATM condition with OE revealed trivial effect sizes for 4 parameters out of 5 and a small effect size for the ML oscillations. Similarly, LTM condition with OE showed small effect sizes for all the 5 parameters. In the CE condition, the effect sizes were trivial for all the parameters excluding ellipse area, sway area, and ML oscillations in LTM condition. The explanation of these low effect sizes could be attributable to the running performed below the estimated anaerobic threshold. Indeed, Nardone and colleagues reported how this intensity little affected body sway with respect to more strenuous efforts (Nardone et al., 1997). Moreover, the slight increase of effect sizes in the LTM with respect to the ATM could be explained by the higher linear head accelerations occurring while running in LTM. In fact, higher head accelerations could have decreased the sensibility threshold of the otholitic system not only during running but also at the beginning of the recovery time (Lepers et al., 1997). Therefore, we can conclude that in the prescription of physical exercise at moderate intensity, the choice of ATM or LTM treadmill is not determinant concerning the short-term effects on postural balance performance.

### Postural control mechanisms

SDA analysis allowed studying the human postural control mechanisms assuming that the maintaining of the erect posture could be viewed as a stochastic process (Collins and De Luca, 1993). From a motor control perspective over short-term intervals open loop control strategies are adopted while over long-term intervals closed loop control strategies are involved (Collins and De Luca, 1993). Diffusion coefficients represented an objective measurement of the postural instability considering the ML (*Dfx*) and the antero-posterior (*Dfy*) axis as well as the plane of support (*Dfr*^2^): a higher value of these coefficients reflected a less tightly regulated control system (Collins and De Luca, 1993). The main finding of the statistical analysis concerns the effect of the treadmill factor on all the short-term diffusion coefficients (*Dfxs, Dfys, Dfsr*^2^*)* in the OE condition. Thus, treadmill seems to negatively influence the open loop motor control strategies where corrective feedback mechanisms are still not called into play (Collins and De Luca, 1993). As for the classical postural parameters, in the CE condition, only a treadmill effect (*Dfxs)* and a time effect *(Dflr*^2^*)* were detected. Again these findings could be explained by the fact that treadmill running negatively affects visual contribution to static postural stability (Derave et al., 2002). We can speculate that the visual deprivation can be mitigated by the interventions of the other postural control systems. Once again to quantify the relevance of our results it has to be taken into account the size of the differences. About that, all effect sizes ranged from trivial to small. Therefore, as for the postural balance performance, the functional organization of the postural control mechanisms is slightly influenced both by the moderate intensity of the running and by the type of treadmill employed. Moreover, this influence occurred in the OE condition and referred to the short-term intervals where open loop control mechanisms are employed and corrective feedback mechanisms are not called into play.

To deepen the understandings of postural control mechanisms we further considered the time-development of the subsequent COP trajectories as an outcome of random and non-stationary fluctuations in the postural control system (Roerdink et al., 2006). The SampEn algorithm allowed to calculate the regularity of a signal and has been already applied to postural sway data (Roerdink et al., 2006; Ramdani et al., 2009). Moreover, the regularity of a physiological time-series is representative of the efficiency of its control system (Goldberger et al., 2002). An overall higher regularity (i.e., lower SampEn values) of the postural balance control was detected in the CE condition. It has been demonstrated that the more regular the COP displacements, the greater the amount of attention invested in postural balance control (Donker et al., 2007). We can speculate that for our subjects standing with eyes opened did not require the same level of attention as was employed in the CE trials. Certainly, removal of visual cues amplified the consciousness of postural balance (i.e., attention to an internal focus), leading to a voluntary control (Hunter and Hoffman, 2001; Andersson et al., 2002). Moreover, the loss of complexity of physiological and behavioral systems occurs when the number of the structural components is reduced and/or their interaction is altered (Ramdani et al., 2009). Although the highlighted effect sizes were small, our findings underlined an increase of the AP and ML SampEn after LTM running in the CE condition. This could be related to a functional decline of the postural balance control which resulted in maladaptive responses consequent to LTM running (Hansen et al., 2017). Moreover, we can speculate that a higher LTM running perturbation could result in an inappropriate attentional involvement of the postural control system (Schniepp et al., 2013).

## Conclusions

Results of the present study underlined how running at moderate intensity on ATM and LTM slightly altered postural balance performance as well as the relative postural control mechanisms. However, trivial and small effect sizes weakened the relevance of the described alterations. Therefore, the usage of ATM should be taken into account in all those situations where the well-known advantages of the aquatic environment are priorities. Namely, reducing joint loads (Greene et al., 2009; Kanitz et al., 2015), improving the recovery from exercise (Wilcock et al., 2006), and decreasing the HR (Barbosa et al., 2007). Nonetheless, some considerations have to be acknowledged. Firstly, we studied a group of young healthy participants, and thus these results might not necessarily reflect unhealthy or elderly subjects. Secondly, our experimental protocol consisted of an acute exposure to aquatic and land treadmill (LTM). Hence, our findings could motivate further studies on ATM employment among frail elderly populations. Indeed, if the unaltered postural balance control will be confirmed, ATM would be a good safety choice against fall-risk and an aquatic environment would be beneficial for the musculoskeletal and cardiovascular system.

## Ethics statement

The study conformed to standards for the use of human subjects in research as outlined in the current declaration of Helsinki and was approved by the ethical board of the Department of Biomedical Sciences of the University of Padova. Subjects involved in the study read and signed an informed consent.

## Author contributions

GM, AR, AP, and GB conceived and designed the experiments. AR, GZ, and GM performed the experiments. AR, GM, MB, and AC analyzed the data. AC and AP contributed materials. GM, AR, and MB wrote the paper. All authors approved the final version of the manuscript.

### Conflict of interest statement

The authors declare that the research was conducted in the absence of any commercial or financial relationships that could be construed as a potential conflict of interest.

## Abbreviations

LTM: Land treadmill
ATM: Aquatic treadmill
SampEn: Sample entropy
OE: Opened eyes
CE: Closed eyes
CoP: Center of pressure
AP: Anterior-posterior
ML: Medio-lateral
SDA: Stabilogram diffusion analysis
*Dfxs*, Short term diffusion coefficient along the x axis*; Dfxl*: Long term diffusion coefficient along the x axis
*Dfys*: Short term diffusion coefficient along the y axis
*Dfyl*, Long term diffusion coefficient along the y axis*; Dfr*^2^*s*, Short term diffusion coefficient combining x and y axis*; Dfr*^2^*l*: Long term diffusion coefficient combining x and y axis.
